# Surrogate skin-to-skin care: the “donor milk” of kangaroo mother care

**DOI:** 10.1038/s41372-025-02538-0

**Published:** 2025-12-22

**Authors:** Itamar Nitzan, Raylene Phillips, Robert D. White, Mario Rüdiger, Elizabeth E. Rogers

**Affiliations:** 1 Department of Neonatology, Shamir Medical Center, Jerusalem, Israel; 2 The Faculty of Medicine, Hebrew University, Jerusalem, Israel; 3 Loma Linda University Children’s Hospital, Loma Linda, CA USA; 4 Loma Linda University School of Medicine, Loma Linda, CA USA; 5 Beacon Children’s Hospital, South Bend, IN USA; 6 Saxonian Center for Feto/Neonatal Health, Faculty of Medicine and University Hospital Carl Gustav Carus, Technische Universität Dresden, Dresden, Germany; 7 Department of Pediatrics, University of California San Francisco, San Francisco, CA USA

**Keywords:** Paediatrics, Health policy

## Abstract

Skin-to-skin care (SSC) significantly decreases mortality and improves preterm infants’ outcomes. The World Health Organization recommends that every preterm baby receive 8–24 h per day of SSC beginning as soon as possible after birth but in many settings this goal is rarely met. An important barrier for SSC is parent availability; lack of parental leave, siblings who require care, and other factors often limit parents’ availability for SSC. In many studies that demonstrated the benefits of SSC, including infection rate reduction, both parents and surrogates participated in SSC. Though not as ideal as parental SSC, surrogate SSC can be compared to donor human milk which does not provide all benefits of mother’s own milk but is considered superior to formula. An analogous benefit could be true for infants who receive less than recommended parental SSC if surrogates support parents in providing extended periods of SSC.

## Introduction

Skin-to-skin care (SSC) of a newborn consists of holding the infant against the bare chest of the caregiver. It has been shown to significantly decrease mortality in preterm infants and to improve numerous physiologic parameters when performed for extended periods [1, 2]. During SSC, preterm infants are more physiologically stable, experience less pain, and require less oxygen supplementation. Preterm infants given SSC also demonstrate improved growth and have fewer infections. Kangaroo Mother Care (KMC) is defined by the World Health Organization (WHO) as “continuous and prolonged skin-to-skin contact of the baby with the mother and support for exclusive breastfeeding or breastmilk feeding” [3].

## Benefits of SSC

A large, randomized trial on immediate KMC (iKMC), a type of early and continuous or prolonged SSC, conducted in five low-income countries showed that beginning SSC for low birthweight infants immediately (or as soon as possible) after birth and continuing it for prolonged periods every day reduced neonatal mortality by 25% [4]. These results prompted the WHO to update their recommendations for the care of low birthweight infants (LBWI) in 2022. Previous guidelines had recommended waiting until after LBWI were stabilized (3–7 days) before beginning KMC, but new recommendations include beginning KMC as soon as possible after birth and performing KMC for 8–24 h per day as much as possible [3]. This update acknowledges what has been known in animal research for decades - that separation from their mother is a highly stressful experience for newborn mammals and that a newborn’s mother is a source of physiological regulation.

Despite the multiple well-documented benefits of prolonged SSC, providing this intervention for infants in the Neonatal Intensive Care Unit (NICU) is challenging. Recently, Lazarus et al. evaluated retrospectively the average daily duration of SSC in a tertiary NICU and its effect on neurodevelopmental outcomes. The authors reported an average daily duration of SSC of 17.76 ± 16.62 min, less than 4% of the minimum WHO recommendation [5].

Andrews et al. conducted a randomized, controlled study of 46 mothers to evaluate the effect of financial incentives on the duration of SSC and found that mothers receiving financial incentives performed on average 43% more time of SSC than mothers not receiving financial incentives [6]. In this study, the average daily SSC duration was 28 min in the control group compared to 44 min in the financial incentive group, which is still less than 10% of the minimum WHO recommendations.

## Challenges of meeting SSC recommendations

In order to increase SSC duration, several systems-level challenges for supporting SSC need to be considered, including safety concerns regarding possible IV line and endotracheal tube dislodgment, limited availability of hospital personnel to support transferring infants safely from beds to chests, inadequate education and training of healthcare workers about the importance of SSC, and how to do it safely.

Another consideration is unit culture or beliefs about SSC benefits and real or perceived barriers. A study by Coutts et al. explored barriers for Kangaroo Care in British Columbia, Canada. Documented barriers included not only the physical environment in the NICU, but also “healthcare providers’ beliefs about kangaroo care,” including their belief in the “local feasibility of Kangaroo Care” becoming an integral part of their NICU routines and their individual comfort level in transferring preterm babies with lifesaving lines and tubes [7].

Efforts have been made around the world to address NICU culture and the perceptions and beliefs of staff about SSC. For example, a QI project performed at a level IV NICU in the USA, incorporated four key drivers for increasing SSC in their unit, three of which addressed staff culture: (1) Multidisciplinary approach, (2) Nurses’ engagement, and (3) Improving NICU culture [8]. This QI project successfully increased SSC more than two fold; but even then, SSC increased only to an average of 32 min per eligible patient day.

NICU culture and staff barriers deserve dedicated research on ways to understand and effectively address them. Many NICU staff are trained on the techniques of *how* to do SSC without providing education about *why* it is so important for the baby’s short-and long-term stability, growth, and development, as well as for the mental health and wellbeing of parents. An important principle of adult learning is motivation. While children often learn because they are told to, adults learn best when they understand the relevance of what they are learning [9]. Learning why doing a new task is important for the wellbeing of their patients provides motivation to learn and perform the techniques of doing it well.

In this article we would like to address parental barriers and a potential way of mitigation. Individual parent barriers for early and prolonged SSC include lack of guaranteed protected parental leave in some countries, other siblings who require care at home, distance from the hospital, lack of transportation, parental medical conditions, fear of complications, and inadequate education regarding the benefits of SSC. Those barriers may be even more common in families from minoritized communities, who have a non-majority language preference, live in rural places, or are single parents, increasing the disparity for families already impacted by the crisis of having an infant in the NICU [10].

Many parents experiencing NICU care have family members or friends who may be willing to act as a volunteer SSC-surrogate and join the parents in being available to perform SSC with their preterm infant in the NICU. This may include grandparents, adult or teenage siblings, or other family and community members. Some parents who do not have extended family support may wish to have specially trained, non-related volunteers to perform surrogate SSC with their infant when the parents cannot be present in the NICU. To help meet the goal of nearly continuous SSC in the landmark iKMC study, all mothers were asked to identify one or two adult women who could act as surrogates in providing KMC [4].

## Theoretical considerations on surrogate SSC

Currently there is limited evidence to show the potential benefits of surrogate SSC; nevertheless, it can be argued that surrogate SSC could be analogous to the provision of donor human milk for preterm infants in the NICU. Donor milk does not provide all the benefits of mother’s own milk (MOM), but it is considered superior to non-human formula [11, 12]. A similar trade-off could be true for infants who are not able to receive prolonged SSC from their own parents but might experience the benefits of more human contact and modulated multisensory stimulation from a surrogate.

When compared with SSC, placing a preterm infant in an incubator causes separation from his/her mother (known to result in significant stress), and hence may increase the risk of sepsis and mortality. Nevertheless, incubator care is needed for extremely preterm infants when no one is unavailable for SSC or in extreme medical circumstances. The iKMC trial showed a 25% reduction in mortality when SCC was increased from averages of 1.5 h per day to 17 h per day in the first week of life. In this study, SSC was done with either the mother or another adult woman chosen by the mother [4]. These findings suggest that depriving preterm infants of the physiological care mode of SSC (with mother or another caregiver) for most of each day during the first week of life results in a 25% higher mortality.

Shonkoff et al. have defined toxic stress for an infant or a child as “strong and prolonged activation of the body’s stress management systems in the absence of the buffering protection of adult support” [13]. Since being in the extra-uterine environment of the NICU is known to cause “strong and prolonged” stress to preterm infants, providing SSC can be considered a form of “buffering protection of adult support.”

To prevent the potentially harmful effects of separation for infants whose parents have limited ability to perform SSC, an alternative is highly warranted. Understanding that SSC benefits both the infant and the parent, an alternative will not be as ideal as their parent but could be more acceptable than being separated from the benefits of human touch and care while in the incubator.

## Data regarding safety and efficacy of surrogate SSC

Several trials have shown improved physiologic parameters in preterm infants with non-maternal SSC. Skin-to-skin contact with either parent has demonstrated a comparable physiologic response in the preterm infant being held [14, 15], as does SSC with either mothers or grandmothers [16]. SSC provided by 1–2 surrogate caregivers was an important component of the iKMC trial where maternal SSC made up ~73% of total SSC, the remainder of which was provided by a surrogate chosen by the mother.

Surrogate SSC is practiced occasionally in many units in the USA and Europe, and to the best of our knowledge there are no publications regarding an increase in infection with this practice. On the contrary, rates of sepsis and suspected sepsis were reduced in the iKMC trial, which included surrogate SSC [4, 17]. Suggested mechanisms in which surrogate SSC may reduce infections may include that the microbiome of a healthy human’s skin (parent or surrogate) may be less dangerous than antibiotic-resistant NICU flora that that have been found to be present in many neonatal incubators [18]. In addition, psychological stress has been shown to alter the immune response in children and adults [19, 20]. Hence, reducing the chronic stress of the NICU environment by the “buffering protection of adult support” in SSC with either a parent or surrogate may improve the baby’s immune system and resilience.

## A suggested framework for surrogate SSC

We suggest that surrogate SSC should be offered and performed in addition to (but not a substitute for) parental SSC. Parents should receive education that because newborns are already familiar with their parents, and because of the parents’ roles in the child’s development, as well as the benefits to parents themselves, parental SSC is significantly better than surrogate SSC. However, because SSC is critically important to the infant’s short- and long-term development and wellbeing, parents should be supported to consider surrogate SSC at times and in circumstances when they are not available. This information will emphasize the importance of SSC and may, actually, increase parental SSC in the same way as providing donor milk emphasizes the importance of human milk for preterm babies and may help increase MOM and breastfeeding rates [21].

Parents should be advised that in an ideal situation, surrogates would have an emotional connection with the cared infant; someone who will be part of the infant’s and child’s life in the future. Studies have found positive comparisons in the effects of SSC with either mothers or fathers and with either mothers or grandmothers. However, to the best of our knowledge, no study has compared physiological parameters of preterm infants in SSC with surrogates who are non-parental family members vs. surrogates who are not related to the baby, or between female vs. male surrogates.

Before surrogates perform SSC, parents should be consulted with and agree to the surrogate providing SSC for their infant and should have input into who and how many surrogates may be included based on their specific situation (e.g., single parents and parents of multiples may benefit from more surrogates). Exclusion criteria for surrogacy should be carefully considered; for example, it is suggested that potential surrogates who have been recently hospitalized, received recent broad spectrum antibiotics, or are feeling unwell may be excluded from performing surrogate SSC.

Parents and non-parental volunteers for SSC all need education about the benefits of SSC for preterm infants as well as training on how to perform it safely and effectively, including transferring the baby from bed to chest, hands and chest hygiene, understanding preterm behavioral cues, and beneficial effects of softly singing or talking directly to the infant. Ideally, each NICU will have this type of basic education for parents who are performing SSC. Non-parental volunteers for SSC should receive at least the same education and training as parents receive, as well as education about their role in helping parents to provide the recommended duration of 8–24 h of SSC each day for their hospitalized preterm infant.

Implementation of surrogate SSC should be coupled with extensive team education about the importance of parental SSC and surrogate SSC as a substitute when parents are unavailable. Care providers should be trained in transferring and caring for high acuity neonates during SSC to a level they will feel comfortable to do so, and resources should be allocated to allow for adequate staff availability. NICU policies should reduce limitations on SSC, ideally allowing SSC 24/7. In addition, effort should be taken to advocate for policies that are conducive to increasing parental accessibility to the NICU when their infant is hospitalized, including paid parental leave, extended leave for parents whose infants are hospitalized in the NICU, and safety net policies such as childcare and consideration of financial support. Local resources for parents could include hospital- or community-provided housing in or near the hospital, compensation for travel and hospital parking, and childcare while parents are in the NICU.

## Future evaluation of surrogate SSC

Implementation of surrogate SSC should be evaluated by rigorous studies that will assess its effect on process-related outcomes such as total SSC duration (including trends of parental vs. non-parental SSC) and duration of hospitalization; short-term outcomes of infection rate, changes in the infant’s microbiome and biomarkers of stress for infants, surrogates, and parents; long-term outcomes of growth and neurodevelopment; and longer-term follow up of child and parental mental health (stress, anxiety, and depression). Differences in these outcomes with different types of surrogates (related vs. unrelated, etc.) should also be studied. This may be a unique opportunity to better understand the mechanism in which SSC, with parents and with other human beings, impacts physiological stability, infant growth and neurodevelopment, parent-infant bonding and attachment, and parental and child mental health.
